# Polydatin Protects Bone Marrow Stem Cells against Oxidative Injury: Involvement of Nrf 2/ARE Pathways

**DOI:** 10.1155/2016/9394150

**Published:** 2015-12-06

**Authors:** Meihui Chen, Yu Hou, Dingkun Lin

**Affiliations:** Research Laboratory of Spine Degenerative Disease, Guangdong Provincial Hospital of Traditional Chinese Medicine, Guangzhou 510120, China

## Abstract

Polydatin, a glucoside of resveratrol, has been reported to possess potent antioxidative effects. In the present study, we aimed to investigate the effects of polydatin in bone marrow-derived mesenchymal stem cells (BMSCs) death caused by hydrogen peroxide (H_2_O_2_), imitating the microenvironment surrounding transplanted cells in the injured spinal cord in vitro. In our study, MTT results showed that polydatin effectively prevented the decrease of cell viability caused by H_2_O_2_. Hochest 33258, Annexin V-PI, and Western blot assay showed H_2_O_2_-induced apoptosis in BMSCs, which was attenuated by polydatin. Further studies indicated that polydatin significantly protects BMSCs against apoptosis due to its antioxidative effects and the regulation of Nrf 2/ARE pathway. Taken together, our results indicate that polydatin could be used in combination with BMSCs for the treatment of spinal cord injury by improving the cell survival and oxidative stress microenvironments.

## 1. Introduction

Among central nervous system (CNS) disorders, spinal cord injury (SCI) is the most devastating and traumatic [1, 2]. 40 cases per million individuals are diagnosed as SCI [3]. Bone marrow-derived mesenchymal stem cells (BMSCs), which possess immunosuppressive properties and the capacity for unlimited self-amplification and for terminal differentiation [4, 5], play a privileged role in ameliorating neuronal damage in CNS disease models including SCI [6]. Cellular replacement with MSCs in different SCI animal models has showed functional recovery [7, 8]. However, attempts to transplant BMSCs into animal and human subjects are hampered mainly due to the poor survival of BMSCs [9]. After being transplanted, BMSCs are facing a complicated environment with risk factors that may lead to cell death including oxidative stress [4, 9, 10]. The increased reactive oxygen species (ROS) resulting in sustained oxidative stress in damaged spinal cord is one of the key factors that challenged the survival of donor BMSCs. BMSCs may unavoidably result in apoptosis under oxidative circumstance. Therefore, drugs with antioxidative effects and antiapoptosis may be crucial for the successful transplantation of BMSCs in SCI [10].

Polydatin (Figure 2(a)), isolated from the roots of* Polygonum cuspidatum*, is widely used in traditional Chinese remedies [11–14]. Polydatin has been shown to protect heart function, prevent the development of diabetic renal fibrosis, and ameliorate Alzheimer's disease due to its multiple pharmacological actions, such as antioxidation, anti-inflammation, immunoregulation, antitumor, and neuroprotection [15–18]. However, the protective activity of polydatin on transplanted BMSCs after SCI is unknown.

In this study, we demonstrated for the first time that polydatin might protect BMSCs against H_2_O_2_-induced apoptosis due to enhancing the resistance of BMSCs against oxidative injury and activate the nuclear factor E2-related factor 2 (Nrf 2)/antioxidant response element (ARE) pathway, which has been reported to have key roles in regulating endogenous antioxidants and phase II detoxification enzymes, suggesting that polydatin could be a promising approach to increase the cell survival in cell replacement therapy for SCI.

## 2. Materials and Methods

### 2.1. Materials

Male Sprague-Dawley (SD) rats (100 ± 20 g) were supplied by the Center of Experimental Animals, Guangzhou University of Chinese Medicine (Guangzhou, China, Certificate number 00100561). All procedures were performed according to animal guidelines of Guangzhou University of Chinese Medicine. Polydatin was purchased in Aladdin (Shanghai, China). Trypsin, 3-(4,5-dimethylthiazol-2-yl)-2,5-diphenyltetrazolium bromide (MTT), dimethyl sulfoxide (DMSO), Hochest 33258, and dichlorofluorescein diacetate (H_2_DCF-DA) were purchased from Sigma-Aldrich (MO, USA). Low-glucose Dulbecco's modified Eagle's medium (LG-DMEM) and fetal bovine serum (FBS) were obtained from Gibco-BRL (NY, USA). Hydrogen peroxide, lactate dehydrogenase (LDH), Annexin V FITC/PI, Cell-Light 5-ethynyl-2′-deoxyuridine (EdU) Apollo594 in vitro Image kit, and glutathione (GSH) assay kits were purchased from Keygen (Nanjing, China). Brusatol was bought from Chengdu PureChem-Standard Co., Ltd. (Chengdu, China). Polydatin (Aladdin) was dissolved in DMSO before dilution with the culture medium. The final concentration of DMSO was 0.1%.

### 2.2. Cell Culture and Treatment

Culture of rat BMSCs was performed as previously described [19]. Briefly, all bone marrow was flushed out with a 10 mL syringe using LG-DMEM supplemented with 10% FBS. The whole marrow washouts were collected, centrifuged, and plated into a culture flask in 37°C under 5% CO_2_. All cells used in the assay were of passages 3–5. The phenotypic properties of BMSCs were identified by flow cytometry as previously reported [20]. Cells were pretreated with polydatin for 2 h and then treated with H_2_O_2_ (600 *μ*M) for 24 h.

### 2.3. Cell Viability Assay

Cell viability was measured by MTT assay. Cells were plated on 96-well plates at a density of 1 × 10^4^ for 24 h. After incubation with H_2_O_2_ for 24 h, 10 *μ*L MTT (5 mg/mL) was then added to each well and the mixture was incubated for 3 h at 37°C. MTT reagent was then replaced with DMSO (100 *μ*L per well) to dissolve formazan crystals. After the mixture was shaken at 37°C for 15 min, absorbance was determined at 570 nm using a microplate reader. Results were expressed as the percentage of MTT reduction and the absorbance of control cells was set as 100%.

### 2.4. LDH Release Assay

The cytotoxicity was measured by LDH release assay. LDH is a cytoplasmic enzyme retained by viable cells with intact plasma membrane and released from cells with damaged membranes. After the indicated treatment of BMSCs, the medium was collected and assayed for LDH activity as previously reported [21]. Briefly, the release of LDH is measured with a coupled enzymatic reaction that results in the conversion of a tetrazolium salt into red-colored formazan, which is correlated with LDH activity. The formazan was measured with a microplate reader at 450 nm. Results were expressed as the percentage of LDH release and the absorbance of control cells was set as 100%.

### 2.5. Hochest 33258 Assay

To detect morphological evidence of apoptosis, cell nuclei were visualized by DNA staining with the fluorescent dye Hochest 33258. After treatment, BMSCs were stained with Hochest 33258 (1 *μ*g/mL) for 15 min in the dark. Results were tested by visual observation of nuclear morphology through fluorescence microscopy (Olympus, Japan) equipped with a UV filter.

### 2.6. Annexin V-FITC Assay

The apoptotic ratios of cells were determined with the Annexin V-FITC apoptosis detection kit. Briefly, BMSCs were collected and washed twice with cold PBS buffer, resuspended in 500 *μ*L of binding buffer, incubated with 5 *μ*L of Annexin V-FITC, conjugated to FITC and 5 *μ*L PI for 15 min at room temperature, and analyzed by flow cytometry I (BD Biosciences). Cells treated with DMSO were used as the negative control.

### 2.7. Measurement of ROS

Intracellular ROS formation was measured using H_2_DCF-DA as reported [22]. Briefly, after treatment, cells were washed with warm PBS three times and then stained with 10 *μ*M H_2_DCF-DA in serum-free medium for 30 min at 37°C in the dark. DCF fluorescence was analyzed by visual observation of cell morphology through fluorescence microscopy equipped with a UV filter.

### 2.8. Detection of Intracellular GSH

Intracellular GSH concentration was tested by a GSH assay kit. By reacting with dithiobis-nitrobenzoic acid, reduced GSH could form a yellow compound, which is quantifiable at 405 nm and is related to the concentration of the reduced GSH. In brief, whole-cell lysate was prepared according to manufacturer's instructions. The basal contents of GSH in control cells were taken as 100%.

### 2.9. Cell Proliferation

The proliferation of BMSCs was tested with EdU assay. BMSCs were planted into 6-well plate, and then cells were allowed to adhere for 24 h. After the treatment, BMSCs were incubated with EdU for 4 h before fluorescent detection. Cells were fixed with 2% paraformaldehyde for 15 min and stained with EdU kit according to the manufacturer's instructions. Finally, cells were placed under a laser-scanning confocal microscope (LSM710, Carl Zeiss, Germany) for image acquisition.

### 2.10. Western Blot Analysis

Western blotting analysis was performed as previously described [22]. In brief, cellular protein was collected and lysed in lysis buffer. The protein concentration was measured using the BCA assay (Keygen, Nanjing, China). Equal amounts of total protein were separated on SDS-PAGE gel and transferred onto the PVDF membranes (Millipore, Billerica, MA). After blocking with 5% skim milk, the membranes were incubated with primary antibodies Bcl-2, Bax, Nrf 2, and NQO-1 (Cell Signaling Technology, Beverly, MA, USA) overnight at 4°C, followed by sequential incubation with horseradish peroxidase-conjugated secondary antibodies for 2 h. The bands were visualized by an enhanced chemiluminescence detection kit (ECL, Amersham Arlington Heights, IL, USA) and exposed to gel imaging system. The intensities of bands were performed using Quantity One Software (Bio-Rad, Hercules, CA).

### 2.11. Statistical Analysis

The data were presented as mean ± S.E.M. Statistical analyses between two groups were performed by unpaired Student's *t*-test. Differences among groups were tested by one-way analysis of variance (ANOVA). A probability value of *p* < 0.05 was accepted to be statistically significant.

## 3. Results

### 3.1. Characterization of BMSCs

**Figure 1:**
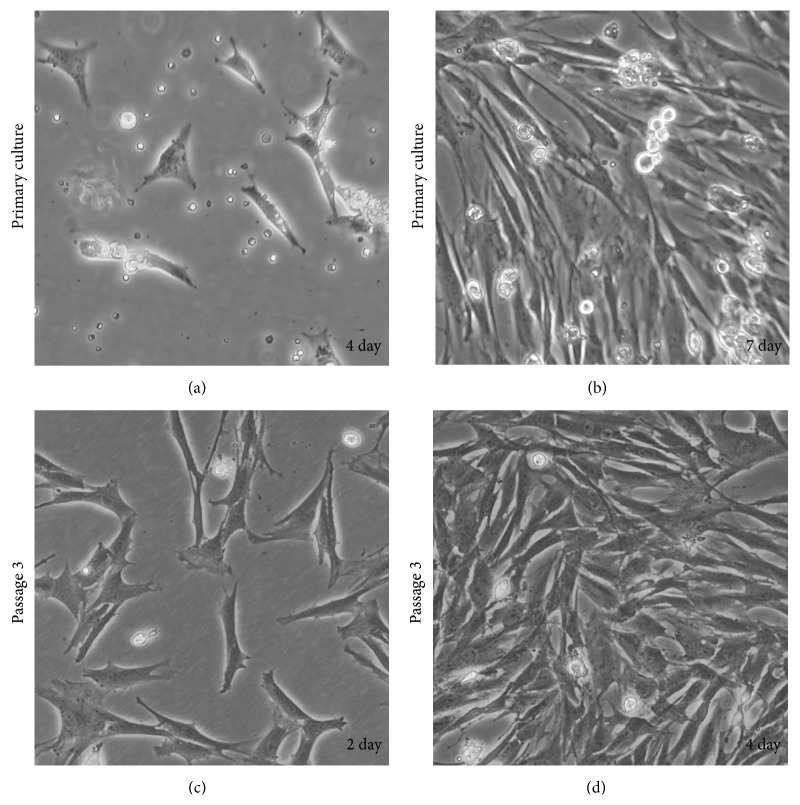
Representative fields of BMSCs morphologies.

BMSCs were isolated from rat bone marrow, expanded in primary culture and passaged for three times. At initial phase, BMSCs of growth contained attached spindle-shaped cells with colonies and floating cells (Figure 1(a)), reaching confluence at day 7 (Figure 1(b)). The floating cells were completely abolished at passage 3 (Figures 1(c) and 1(d)).

### 3.2. Effects of Polydatin on BMSCs Exposed to H_2_O_2_

**Figure 2:**
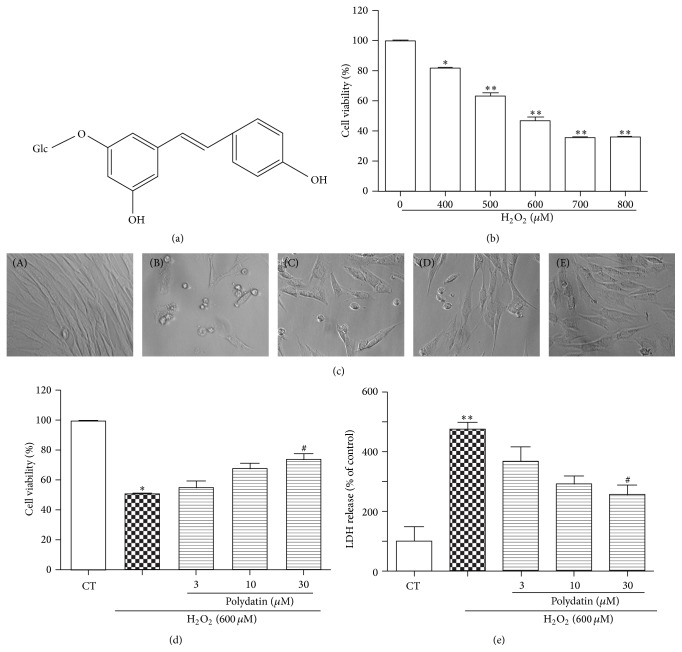
Effects of polydatin on the cell viability of BMSCs exposed to H_2_O_2_. Cells were pretreated with different concentrations of polydatin for 2 h followed with H_2_O_2_ (600 *μ*M) for 24 h. (a) Structure of polydatin. (b) Cells were treated with different concentrations of H_2_O_2_ for 24 h. (c, d) Cell viability was measured by MTT assay and cells were photographed under phase-contrast optics. (A) CT, (B) H_2_O_2_, (C) polydatin 3 *μ*M + H_2_O_2_, (D) polydatin 10 *μ*M + H_2_O_2_, and (E) polydatin 30 *μ*M + H_2_O_2_. (e) Cell death was measured by LDH assay. Bar graph represents independent experiments, each performed in triplicate. One-way ANOVA followed by Tukey's test. Data are presented as means ± S.D. ^*∗*^ *p* < 0.05 and ^*∗∗*^ *p* < 0.01 versus control group. ^#^ *p* < 0.05 versus H_2_O_2_-treated group.

The viability of BMSCs treated with H_2_O_2_, ranging from 400 to 800 *μ*M for 24 h, decreased dose-dependently. 600 *μ*M H_2_O_2_ caused approximately cell death by 50% (Figure 2(b)) and the concentration was chosen for the following experiments. To investigate the effects of polydatin on H_2_O_2_-induced cell death, MTT and LDH assays were applied. The results showed that polydatin significantly increased cell viability (Figures 2(c) and 2(d)) and decreased cell death (Figure 2(e)).

### 3.3. Polydatin Reduced H_2_O_2_-Induced Apoptosis-Like Cell Death

**Figure 3:**
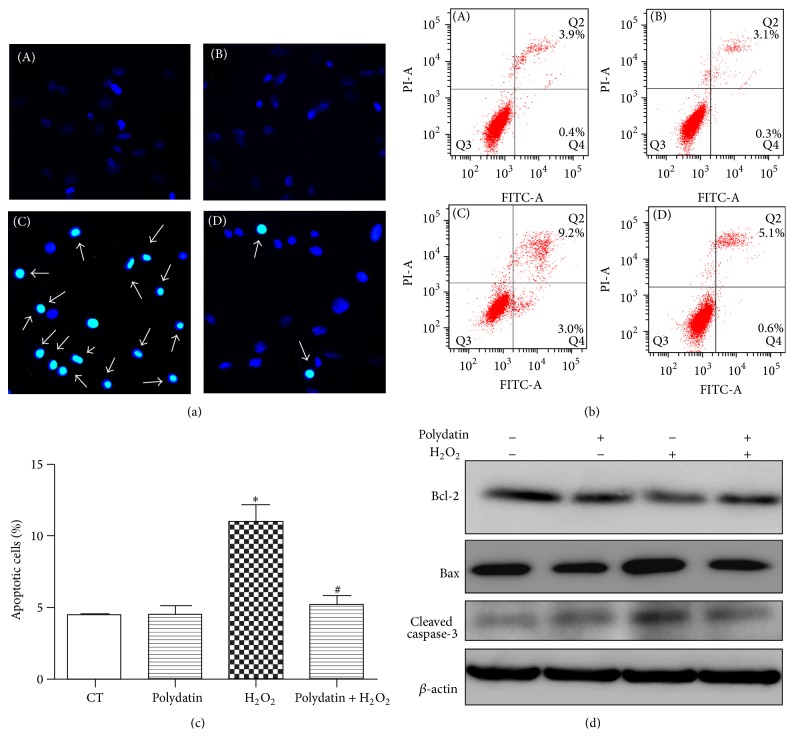
Polydatin attenuated H_2_O_2_-induced apoptosis in BMSCs. (a) Hochest 33258 staining was applied to detect the nuclear condensation of BMSCs, pretreated with polydatin in presence of H_2_O_2_. Fluorescence images (A–D) were observed by fluorescence microscope. (A)–(D) represented CT, polydatin, H_2_O_2_, and H_2_O_2_ + polydatin group, respectively. (b) BMSCs were pretreated with 30 *μ*M polydatin for 2 h and followed by exposing to H_2_O_2_ (600 *μ*M) for 12 h. The induction of apoptosis was determined using Annexin V-FITC/PI staining. (c) Quantitative analysis of apoptotic cells in Figure 3(b). (d) The expression of Bcl-2, cleaved caspase-3, and Bax of BMSCs exposed to H_2_O_2 _with or without polydatin. Data are presented as means ± S.D. ^*∗*^ *p* < 0.05 versus control group; ^#^ *p* < 0.05 versus H_2_O_2_-treated group.

Hochest 33258 staining and Annexin V-propidium iodide (PI) staining assay were used to observe whether H_2_O_2_ induced apoptotic death. Our results showed that H_2_O_2_ induced nuclear condensation (Figure 3(a)), which was blocked by polydatin. The total apoptotic rate (total rate of the cells that are Annexin V positive and PI positive) of H_2_O_2_ group (10.95%  ±  1.25) was significantly increased compared with control group (4.45%  ±  0.15), and polydatin effectively reduced the apoptotic rate (5.15%  ±  0.75) (Figures 3(b) and 3(c)). Furthermore, after treatment with H_2_O_2_, upregulation of proapoptotic protein Bax and cleaved caspase-3 and downregulation of antiapoptotic protein Bcl-2 were observed in BMSCs, which were reversed by polydatin pretreatment.

### 3.4. Polydatin Decreased the Intracellular ROS Formation

**Figure 4:**
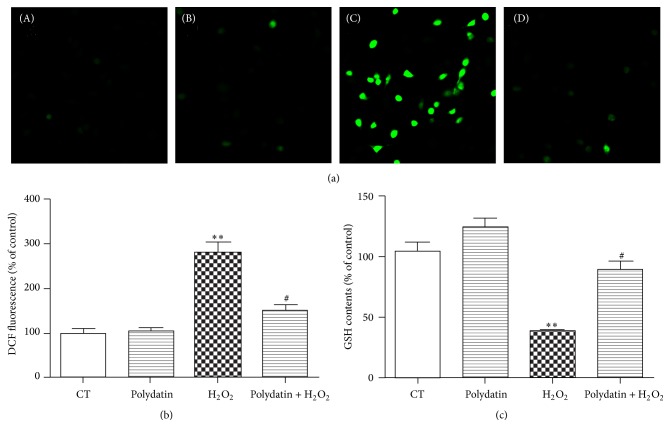
Polydatin scavenges ROS produced by H_2_O_2_. (a) ROS production induced by H_2_O_2_ was detected by H_2_DCF-DA staining. (b) Quantitative analysis of DCF fluorescent intensity. (c) The level of GSH was measured using GSH assay kit. The basal contents of GSH in untreated control cells were taken as 100%. Data are collected from 3 independent experiments and presented as means ± S.D. One-way ANOVA followed by Tukey's test. ^*∗*^ *p* < 0.05 and ^*∗∗*^ *p* < 0.001 versus control group; ^#^ *p* < 0.05 versus H_2_O_2_-treated group.

To further disclosure the protective mechanism of polydatin, we detected its effects on the formation of intracellular ROS by H_2_DCF-DA staining, a ROS probe, and the endogenous antioxidant glutathione (GSH) using a GSH assay kit. As shown in Figures 4(a) and 4(b), compared with the control group, H_2_O_2_-treated group cause significant increase of ROS, which was attenuated by polydatin. Moreover, polydatin also improve the intracellular GSH which was depleted by H_2_O_2_ (Figure 4(c)).

### 3.5. Effects of Polydatin on the Cell Cycle of BMSCs

**Figure 5:**
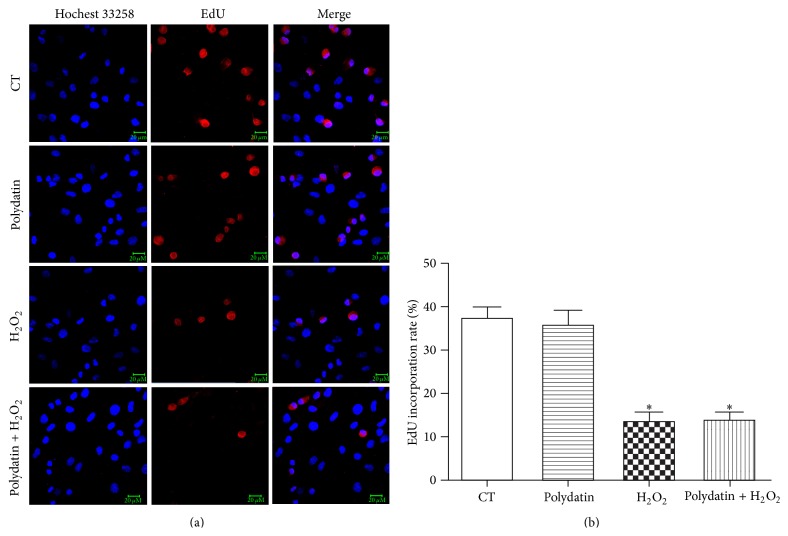
Polydatin did not inhibit the proliferation of BMSCs. (a) Proliferation rate of BMSCs was detected by EdU and Hochest 33258 staining. Fluorescence was visualized by a laser-scanning confocal microscope. Scale bar represents 20 *μ*M. (b) Quantitative analysis of the EdU incorporation rate of BMSCs. ^*∗*^ *p* < 0.05 versus control group.

It is reported that polydatin, the natural precursor of resveratrol, inhibits proliferation of tumor cells caused by the cell cycle arrest [23, 24]. Thus, the survival effect of polydatin indicated in the study might simply be a switch of MSCs into quiescence. To examine whether the protective effects are related to polydatin cell cycle arrest activities, EdU assay was applied. Our results showed that H_2_O_2_ significantly reduced the proliferation rate of BMSCs compared with control group; polydatin at 30 *μ*M did not cause proliferation inhibition on BMSCs, which suggests that polydatin may not lead to cell cycle arrest on BMSCs at the concentration (Figure 5).

### 3.6. Polydatin Prevented BMSCs from H_2_O_2_-Induced Apoptosis through Nrf 2/ARE Pathway

**Figure 6:**
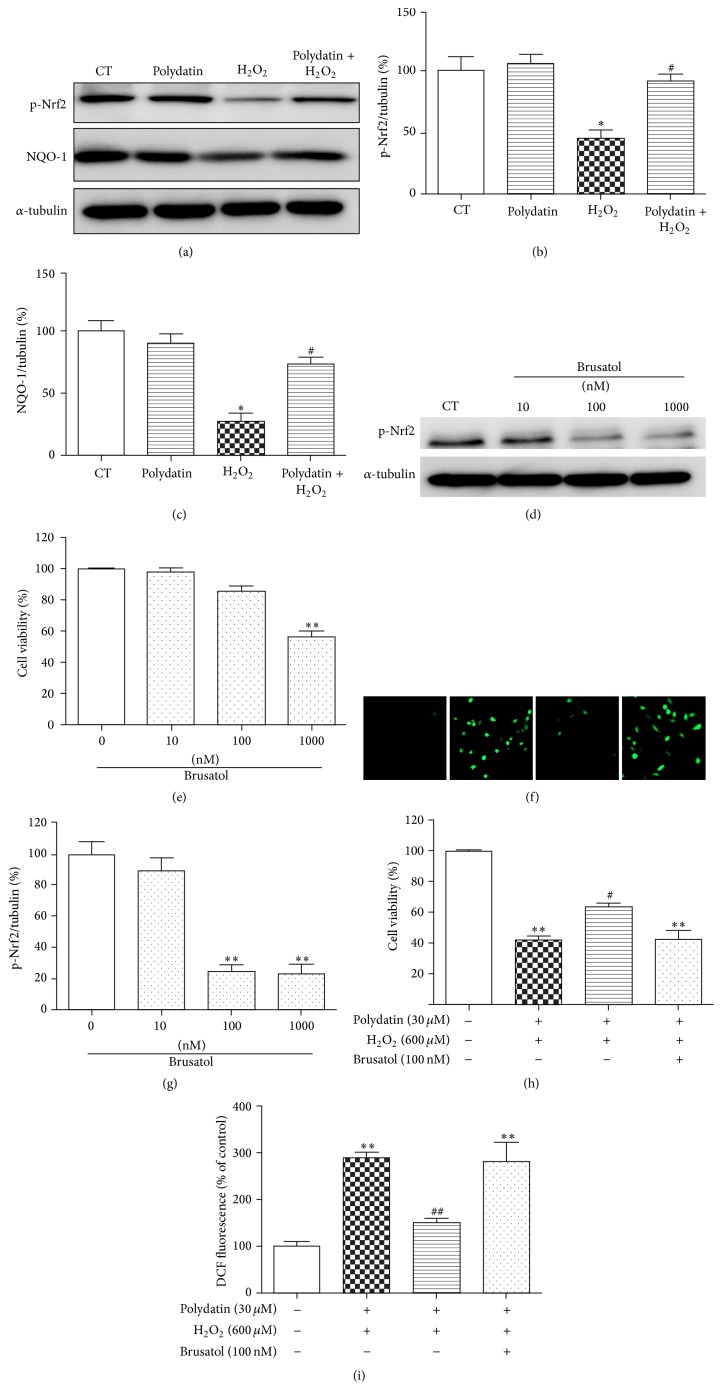
Polydatin protected BMSCs against H_2_O_2_-induced cell death partly through Nrf 2/ARE pathway. BMSCs were pretreated with polydatin for 2 h and further exposed to H_2_O_2_ for 12 h. (a) Effects of polydatin on NQO-1 and the phosphorylation of Nrf 2. (b, c) Quantitative analysis of the blots was shown in panel after being normalized by *α*-tubulin. (d) Cells were treated with different concentration of brusatol for 24 h. Effects of brusatol on phosphorylation of Nrf 2 were detected by Western blot and (g) the bands were normalized by *α*-tubulin. (e) Cell viability was tested in the presence of different concentration of brusatol. (h) BMSCs were pretreated with brusatol (100 *μ*M) for 1 h followed by incubating with/without polydatin and H_2_O_2_ for 24 h. (f) ROS production was detected by H2DCF-DA staining. (b) Quantitative analysis of DCF fluorescent intensity. One-way ANOVA followed by Tukey's test. ^*∗*^ *p* < 0.05 and ^*∗∗*^ *p* < 0.001 versus control group; ^#^ *p* < 0.05 and ^##^ *p* < 0.01 versus H_2_O_2_-treated group.

Polydatin has been reported to quench ROS overproduction by activating Nrf 2/ARE pathway, which has been reported to have key roles in regulating a battery of endogenous antioxidants and phase II detoxification enzymes, including NAD(P)H quinone oxidoreductase-1 (NQO-1) [25]. To explore whether Nrf 2/ARE pathway was involved in the protection of polydatin against oxidative injury, Western blotting was applied. As shown in Figures 6(a)–6(c), H_2_O_2_ significantly decreased the protein levels of p-Nrf 2 and NQO-1 protein, which was partly reversed by polydatin.

To further confirm the involvement of Nrf 2/ARE pathway in the protective effects of polydatin, brusatol, a unique inhibitor of the Nrf 2 pathway, which selectively reduces the protein level of Nrf 2 through enhanced degradation and ubiquitination of Nrf 2, was applied [26, 27]. As shown in Figure 6(d), brusatol at 100 nM significantly reduced phosphorylation of Nrf 2 and did not cause cell death in BMSCs. Therefore, the concentration was chosen for the next experiment. Our results showed that polydatin attenuated cell viability decrease caused by H_2_O_2_, which was reversed by brusatol (Figure 6(h)). Moreover, brusatol also blocked the ROS scavenging activities of polydatin (Figures 6(f) and 6(i)).

## 4. Discussion

To the best of our knowledge, this is the first report about the effects of polydatin on the oxidative injury induced by H_2_O_2_ in BMSCs. We observed that polydatin dramatically attenuated H_2_O_2_-induced ROS generation, GSH depletion, LDH release, and subsequent cell death. Further studies showed that polydatin also enhanced phosphorylation of Nrf 2 and upregulation of NQO-1 which was downregulated by H_2_O_2_, suggesting that polydatin might protect BMSCs against H_2_O_2_ partly via Nrf 2/ARE pathway.

BMSCs, which are capable of self-renewal and differentiation into a variety of mesodermal cell lineages, including osteocytes, chondrocytes, myoblasts, and adipocytes [28, 29] are considered as an ideal source of cells for cell replacement therapy. BMSCs transplantation has shown great promises for treating vast CNS disorders, including SCI. However, poor viability of transplanted BMSCs in injured spinal cord has limited the therapeutic efficiency. Oxidative stress is one of the key mechanisms underlying the pathogenesis of CNS disorders including SCI. Sustained oxidative stress could reduce the survival of donor BMSCs, causing limited reparative capacity of BMSCs. Therefore, it is rational to improve the poor oxidative environment and protect the BMSCs against oxidative stress for the successful transplantation of BMSCs in SCI.

Polydatin, an active stilbene compound isolated from the roots of* Polygonum cuspidatum* Sieb. and Zucc., has been shown to prevent the development of diabetic renal fibrosis, ameliorate Alzheimer's disease, and protect ischemia/reperfusion damage in heart and diabetic nephropathy. It has also been reported to have antiapoptosis and antioxidation activities in many cellular systems. However, protective effects of polydatin on BMSCs are unknown. We used H_2_O_2_ to induce oxidative injury on BMSCs, imitating the poor microenvironment of the spinal cord after SCI. Our results showed that H_2_O_2_ reduced cell viability of BMSCs dose-dependently and caused a robust ROS generation and GSH depletion as previously reported [10]. Polydatin, at a concentration of 30 *μ*M, effectively suppressed H_2_O_2_-induced cell death, scavenged the ROS, and reversed the depletion of GSH, indicating that polydatin exerts beneficial effects on BMSCs as well.

Bcl-2 and Bax are two members of the Bcl-2 family, which are crucial regulatory factors in apoptosis. Bcl-2, the antiapoptotic protein, inhibits apoptosis by preventing cytochrome c release into the cytoplasm [22], while Bax, the proapoptotic protein, promotes apoptosis by inducing mitochondrial membrane depolarization. The Bcl-2 family maintains mitochondrial stabilization by mediating the Bcl-2/Bax balance [30]. Caspase-3 is a pivotal executioner caspase, which triggers the cleavage of a number of proteins and ultimately leads to DNA fragmentation, and has long been considered as a key protease involved in cell apoptosis [31]. In the present study, we examined the underlying mechanism of the protection of polydatin against H_2_O_2_-induced apoptosis by detecting the expression of apoptosis-related proteins using Western blot. We observed the upregulation of Bax and cleaved caspase-3 and downregulation of Bcl-2 following treatment of H_2_O_2_, which were overtly reversed by polydatin, suggesting its antiapoptotic effects.

It is well established that polydatin (also named piceid) and resveratrol inhibit proliferation of tumor cells caused by the cell cycle arrest [23, 24]. Thus, the survival effect of polydatin indicated in the study might simply be a switch of MSCs into quiescence. To examine whether the protective effects are related to polydatin cell cycle arrest activities, we detected the proliferation rate of BMSCs pretreated with polydatin in the presence or absence of H_2_O_2_ using EdU assay. The results showed that polydatin at 30 *μ*M did not cause proliferation inhibition on BMSCs, which suggests that polydatin may not lead to cell cycle arrest on BMSCs at the concentration. According to Su et al., polydatin induced the cell cycle arrest in the S phase at 300 *μ*M on MDA-MB-231 cells but not MCF-7 cells and HepG2 cells, suggesting that polydatin only cause proliferation inhibition in certain cell lines at proper concentrations [23]. According to Su et al., polydatin protected MDA-MB-231 cells against H_2_O_2_ toxicity at 50 *μ*M, a concentration which did not cause cell cycle arrest, indicating that the protective effects of polydatin were independent from its effects on cell cycle arrest. Therefore, the protective effects of polydatin reported in our paper may be just related to its antioxidative activities.

Nrf 2, a basic leucine zipper transcription factor, is reported to drive transcription of all kinds of genes involved in combating products of oxygen radicals and oxidation such as protein and DNA adducts from carbonyls or malondialdehyde [25, 32]. Under normal conditions, Nrf 2 binds to Kelch-like ECH associated protein-1 (Keap1) [33]. When oxidative stress occurs, Nrf 2 is released from Keap1, is translocated to the nucleus, is bound with ARE sequences, and results in transcriptional activation of antioxidant genes including NAD(P)H quinone oxidoreductase-1 (NQO-1) [34]. Huang et al. have reported that polydatin activated Nrf 2/ARE pathway in glomerular mesangial cells [16]. Herein, we found that H_2_O_2_ downregulated NQO-1 and the phosphorylation of Nrf 2 which was partly reversed by polydatin. To further confirm the involvement of Nrf 2/ARE pathway in the protection of polydatin, brusatol was applied. Previous studies reported brusatol as a unique inhibitor of the Nrf 2 pathway, which selectively downregulates the protein level of Nrf 2 via increasing ubiquitination and degradation of Nrf 2 [27]. Herein, we proved that coincubation with polydatin and brusatol reversed the protective and the ROS scavenging effects of polydatin, suggesting that Nrf 2/ARE pathway was involved in the protection and antioxidation of polydatin against H_2_O_2_-induced cell death.

## 5. Conclusion

Taken together, our results indicate that polydatin exerts strikingly protective effects against H_2_O_2_-induced cytotoxicity in BMSCs through activating the Nrf 2/ARE pathway, suggesting that polydatin could be a promising approach to increase the cell survival in cell replacement therapy for SCI.
